# Supplementary material for: Resolution of physics and deep learning-based protein engineering filters: A case study with a lipase for industrial substrate hydrolysis
Source: PLoS One. 2025 Sep 12;20(9):e0332409. doi: 10.1371/journal.pone.0332409 (PMC12431253; doi:10.1371/journal.pone.0332409)
Supplement: S1 File — (DOCX) [file pone.0332409.s001.docx]

**Resolution of physics and deep learning-based protein engineering filters: A case study with a lipase for industrial substrate hydrolysis**

*Spencer Gardiner, et. al*

# Supplementary Materials

Table of Contents

[Supplementary Materials 1](#_Toc196905092)

[S1 preparation of the LipA and substrate structure 1](#_Toc196905093)

[S2 Generation of CONCOORD ensemble 1](#_Toc196905094)

[S3 Modeling of mutations 1](#_Toc196905095)

[S4 Energy minimizations 2](#_Toc196905096)

[S5 Calculation of the PSSM 2](#_Toc196905097)

[S6 Selection and ranking of mutations 2](#_Toc196905098)

[References 7](#_Toc196905099)

## S1 preparation of the LipA and substrate structure

According to the CPD standard model the design protocol required a model of the transition state of the substrate and stabilizing interactions, as well as an enzyme template as a starting structure. Here, LipA was selected for which a crystal structure in its activated state with a co-crystallized substrate is available in the PDB. The start structure of the LipA:Roche ester complex was modeled with SPARTAN (1). The crystal structure of LipA (PDB code 1EX9) solved at a resolution of 2.54 Å is a complex containing 1,2-dioctylcarbamoyl-glycero-3-O-p-nitrophenyl octylphosphonate as a covalently bound substrate analog (2). We used the geometry of this complex structure as a template for modeling the transition state geometry of the Roche ester. The AMBER99SB-ILDN forcefield was chosen for calculations in GROMACS. Because this forcefield does not natively support the Roche ester, the force field parameters including the partial charges were added to the forcefield. For this, two new amino acids were defined: 1) ROC, a representation of the ground state Roche ester, 2) SEO, a representation of the transition state geometry formed with the Roche ester covalently bound to S82. In order to obtain correct partial charges for the Roche ester, a DFT ground state optimization was calculated with SPARTAN (3), using the RB3LYP method and basis set 6-31G(D) (see Fig. 9A). In addition, partial charges for the transition state geometry were obtained for the set of atoms represented in Figs. 9B and 9C. In this figure, the Roche ester is shown covalently bound to the O_γ_ atom of S82. Also, the H251 side chain and a part of the backbone of M16, whose NH group forms stabilizing hydrogen bonds with oxygen atoms of the Roche ester carboxyl group, are included. The charges obtained with SPARTAN were scaled to be compatible with the AMBER charges. For this purpose, a DFT ground state optimization of serine was calculated using SPARTAN, the RB3LYP method and basis set 6-31G(D). The obtained partial charges for serine were compared to the entry in aminoacid.rtp of serine in AMBER99SB-ILDN. The ratio of the SPARTAN values and the AMBER values was used as a factor to scale the ground state and transition state partial charges of the Roche ester. In retrospect, a better compatibility with the AMBER force field might have been achieved by using a combination of Gaussian and RESP charge fitting with Antechamber (4).

## S2 Generation of CONCOORD ensemble

The ensemble-based design protocol to optimize activation energies is illustrated in Fig. 8. It is an iterative protocol that starts with a substrate and crystal protein structure. From the starting protein structure 95 conformations were generated with CONCOORD. In the starting structure, atoms modeled in the transition state conformation were set to occupancy zero to keep them fixed during structure generation. The OPLS all-atom parameters (5) and CONCOORD default parameters were used. We used OPLS instead of AMBER, because the additional substrate constraints did not interoperate with AMBER in CONCOORD as available at that time. These additional constraints made it necessary to iterate up to 5000 times per conformation. The command line prompt was: disco -n 95 -op 1ex9 -i 5000 -ox traj.xtc -or rmsd -of b_factors -con 1000 -t 100 -bump. The starting structure together with the ensemble of 95 conformations yield a final set of 96 structures for further processing.

## S3 Modeling of mutations

Mutations were introduced to each structure of the ensemble through the tool SCWRL4.0 (6). This was achieved by exchanging the original amino acid letter codes with a mutation in the ASCII string representing the amino acid sequence of LipA. The new sequence was loaded along with the coordinate file into SCWRL4.0. All characters in the input string were left in lower case to avoid sidechain optimizations. This was repeated for each structure in the ensemble to create a mutated ensemble. Each structure of the ensemble was prepared for energy minimization in two ways. First, the structure and the Roche ester ground state coordinates (ROC) were merged, which yielded the structures of the reactant state. Second, the residue S82 was replaced with the transition state geometry to construct the transition state structure. The bond, angles, and dihedrals for the residues ROC and SEO were added to the topology files. Inclusion of the ground and transition state geometries does not require docking, because the ensemble was created around the constraints of the substrate present in the desired conformation.

## S4 Energy minimizations

The protein-substrate interactions were calculated with the molecular modeling software GROMACS (7). A necessary prerequisite for the energy calculations was the parameterization of the Roche ester through DFT(8) calculations with SPARTAN, as described above. Energy minimizations were carried out on the ensemble of structures created with CONCOORD. The minimizations were started in implicit GBSA solvent(9) to reduce energy fluctuations from explicit water interactions and to maximize simulation throughput. A steepest decent energy minimization was calculated for 1000 steps in the AMBER99SB-ILDN force field for each of the 96 structures in both, the [ES] and [ES*] ensembles and the final potential energy values $E_{ES,i}$ and $E_{ES*,i}$, respectively, were stored for further processing.

A better forcefield for small molecules would likely enhance the performance of this procedure (10). Furthermore, the calculation of energy differences is only approximate due to the need of computational feasibility. For each mutation 192 simulations had to be calculated. It was found that energy minimization in implicit water is the most feasible approach to allow for sampling of a large mutation-space. In addition, the final ranking and selection of most promising mutants followed a very simple heuristic. It would further be advantageous to compare simulated ${\Delta E}$ values of many more mutations with experimentally determined values to optimize the ranking scheme.

## S5 Calculation of the PSSM

The PSI-BLAST website was used to identify homologous sequences with an expect threshold of 10, a word size of 2, using the BLOSUM62 matrix with gap costs defined as "existence: 11, extension:1" and conditional compositional score matrix adjustment. No filters or masking were used, and the psi-blast threshold was set to E-value = 0.001. In total 1046 sequence homologs were found and used after one iteration to build the PSSM.

## S6 Selection and ranking of mutations

Mutant selection began with calculating ∆Ẽ_v_ values for all possible single-point mutations within 50 residues of the active site. Energy minimizations converged for 669 out of 950 mutations, revealing a distribution with a peak at ∆Ẽ_v_ = 1, indicating many mutations had negligible effects on [ES*] stability. About 40% of mutations lowered ∆Ẽ_v_ (< 1), suggesting potential activity enhancement. From these, 27 variants with ∆Ẽ_v_ below one standard deviation (∆Ẽ_v_ < 0.985) were shortlisted for further screening.

To refine this set, a position-specific scoring matrix (PSSM) filtered out destabilizing mutations. PSSM values were calculated from a PSI-BLAST alignment of 1046 lipase homologs, scoring mutation frequencies across evolutionary sequences (11-13). Mutations with PSSM < -4.0 were excluded, reducing the set to 15 variants. Next, a distance criterion retained only mutations within 5 Å of the substrate, yielding 9 final candidates.

These variants were ranked using two Z-scored metrics. First, the standard deviation (σ_v_) of transition state energy differences (∆E_i_) across 96 ensemble structures quantified transition state entropy. Lower Z_σ_ values indicated mutants that stabilized [ES*] across conformations. Second, the absolute ground-state energy difference (∆E_ESv_) relative to wild-type was averaged over the ensemble and Z-scored, with lower Z_ES_ values indicating stronger substrate binding. The final ranking score was the sum of Z_σ_ and Z_ES_, where lower scores indicated mutants more likely to enhance LipA activity through stabilized transition states and improved substrate affinity (see Supplementary Table S1 for statistics on evaluated mutants).

**Figure S1** *(a) Histogram of ∆E values for 669 single-point mutations of LipA. Mutations with ∆E below 0.985 are highlighted in red. The inlet Venn-Diagram illustrates how additional cutoffs reduce the number of favorable mutations to a final set of 9. (b) A scatterplot of ∆E values against the probability that a certain mutation is found in nature, as expressed in a position specific substitution matrix (PSSM calculated from 1046 sequence homologs). Points that fulfil selection cut-offs ∆E < 0.985 and PSSM >= -4.0 in red. (c) Scatterplot of ∆E values against the distance between mutated residue and the substrate. Points closer than 5 Å to the active site and ∆E < 0.985 in red. (d) Four mutational sites identified with the described protocol. The protein backbone of LipA is shown in cartoon representation. The stick representations highlight the position of Roche ester in LipA as well as the four main mutational hotspots H14, M16, L17, and H81.*

**Table S1:** Summary of all calculations for the LipA + Roche ester complex.

|  | Number of mutations tested/successful | Number of structures in ensemble^1^ | Number of  ensembles  [ES] and [ES*] state | Total number of successful calculations^2^ |
| --- | --- | --- | --- | --- |
| 1 point | 950/669 | 96 | 2 | 128.448 |
| 2 points | 3090/2696 | 96 | 2 | 516.480 |

^1^For each state, ES and ES*, ensembles of 96 structures were energy minimized.

^2^A calculation is classified as successful if at least 90 minimizations converged.

**Figure S2** Multiple Sequence Alignment for LipA obtained from PSIBLAST search with NR database and E-value of 0.001.

170

130

95

72

55

43

34

26

15


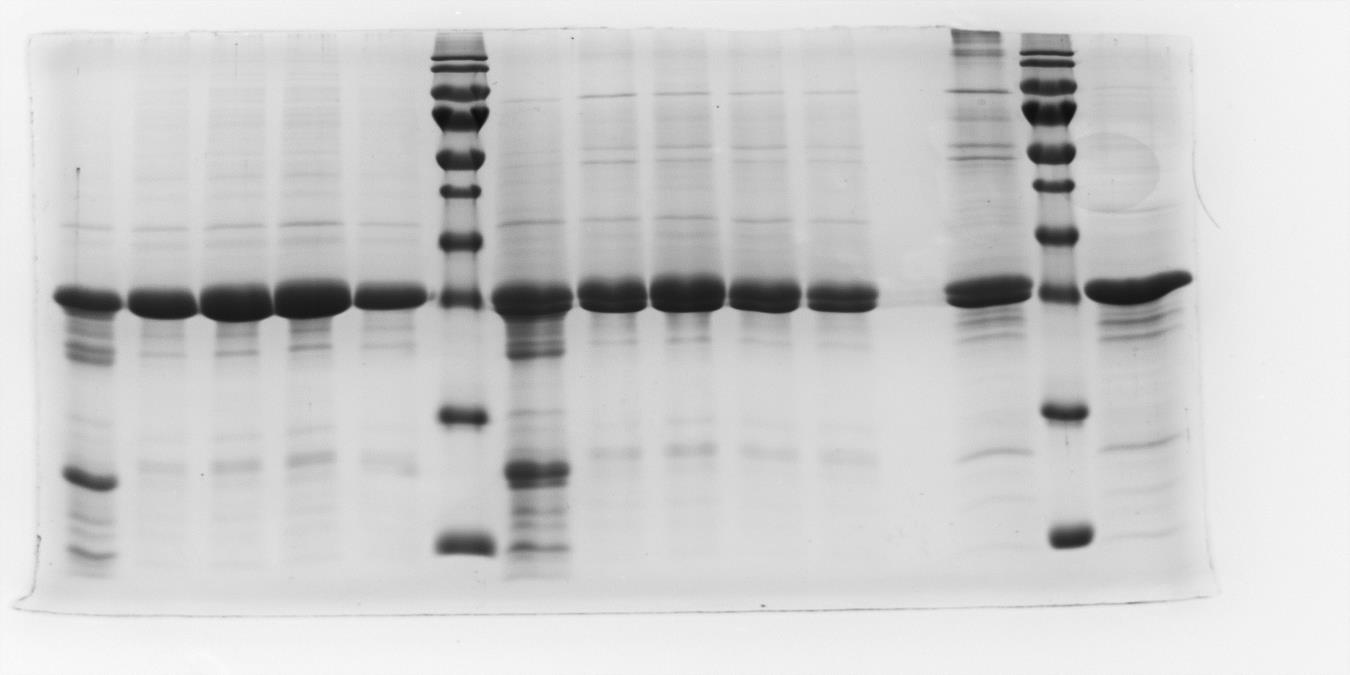


St. M16T M16A L17G H81S H14G


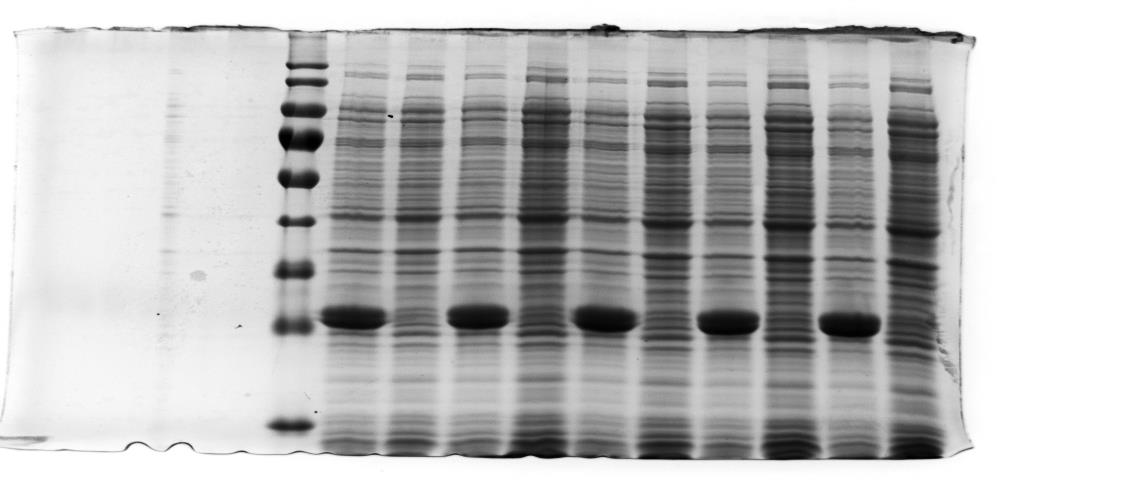


M16T M16A L17G H81S H14G

IPTG St. + - + - + - + - + -

170

130

95

72

55

43

34

26

15

**Figure S4** LipA purified from inclusion bodies. Proteins were analyzed by SDS–PAGE and stained with Coomassie Brilliant Blue G-250. The molecular weights of protein standards (St) are indicated on the right in kDa.

**Figure S3** LipA overexpression. LipA wild-type and mutants were overexpressed in E. coli BL21(DE3) (LB medium, 37°C, 2h induction with 0.4 mm IPTG). Cells equivalent to 100 µl of culture with an OD_580 nm_ of 1 were analyzed by SDS–PAGE and stained with Coomassie Brilliant Blue G-250. The molecular weights of protein standards (St) are indicated on the right in kDa.


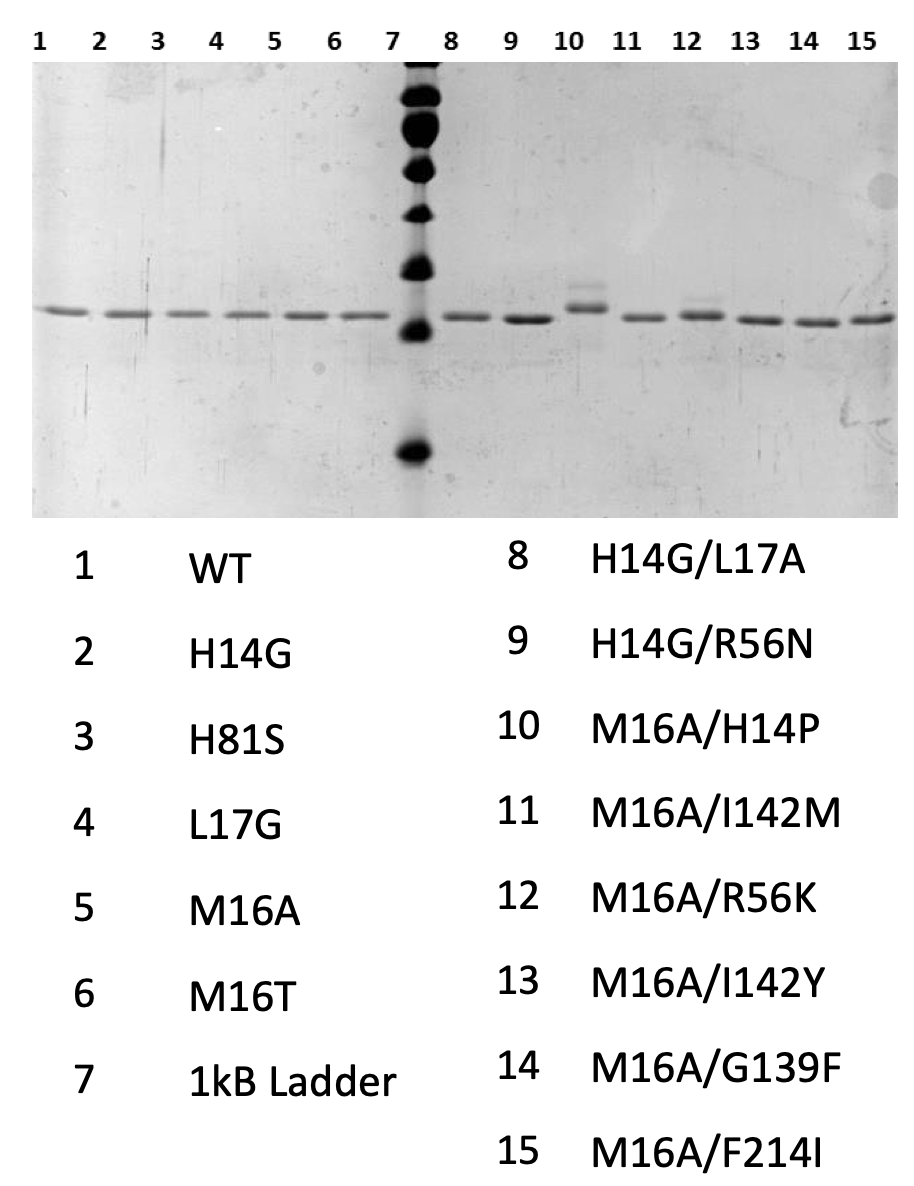


1 2 3 4 5 6 7 8 9 10 11 12 13 14 15

**Figure S5** Purified and activated single and double LipA variants used for enzyme activity measurements. Proteins were analyzed by SDS–PAGE and stained with Coomassie Brilliant Blue G-250. The molecular weights of protein standards (St) are 130, 95, 72, 55, 43, 34, 26, 15 kDa. 1, wild type LipA; 2, H14G; 3, H81S; 4 L17G; 5, M16A; 6, M16T; 7, St; 8, H14G/L17A; 9, H14G/R56N; 10, M16A/H14P; 11, M16A/I142M; 12, M16A/R56K; 13, M16A/I142Y; 14, M16A/G139F; 15, M16A/F214I.

| **Mutation** | **Oligonucleotide sequence (5' 🡪 3')** | |
| --- | --- | --- |
|  | Forward | Reverse |
| H14G | gccgagcatgccgccggccagcacgatg | catcgtgctggccggcggcatgctcggc |
| M16A | gtcgaagccgagcgcgccgtgggccagc | gctggcccacggcgcgctcggcttcgac |
| M16T | ctggcccacggcacgctcggcttcg | cgaagccgagcgtgccgtgggccag |
| L17G | ggcccacggcatgggcggcttcgacaac | gttgtcgaagccgcccatgccgtgggcc |
| H81S | cccgccgtggctgctgccgatcaggttg | caacctgatcggcagcagccacggcggg |
| L17A | ggcccacggcatggccggcttcgacaac | gttgtcgaagccggccatgccgtgggcc |
| R56N | gacacctcggaagtcaacggcgagcagttgct | agcaactgctcgccgttgacttccgaggtgtc |
| H14P | cgtgctggcccccggcatgctcg | cgagcatgccgggggccagcacg |
| I142M | cctcggcgcgctgatgagcttcctttc | gaaaggaagctcatcagcgcgccgagg |
| R56K | ggacacctcggaagtcaagggcgagcagttgctgc | gcagcaactgctcgcccttgacttccgaggtgtcc |
| I142Y | cagcctcggcgcgctgtatagcttcctttccagcg | cgctggaaaggaagctatacagcgcgccgaggctg |
| G139F | gctggtcaacagcctcttcgcgctgatcagcttc | gaagctgatcagcgcgaagaggctgttgaccagc |
| F214I | cgagcgacgccatcctcggcgcc | ggcgccgaggatggcgtcgctcg |

**Table S2** *Oligonucleotides used for the generation of LipA variants.*


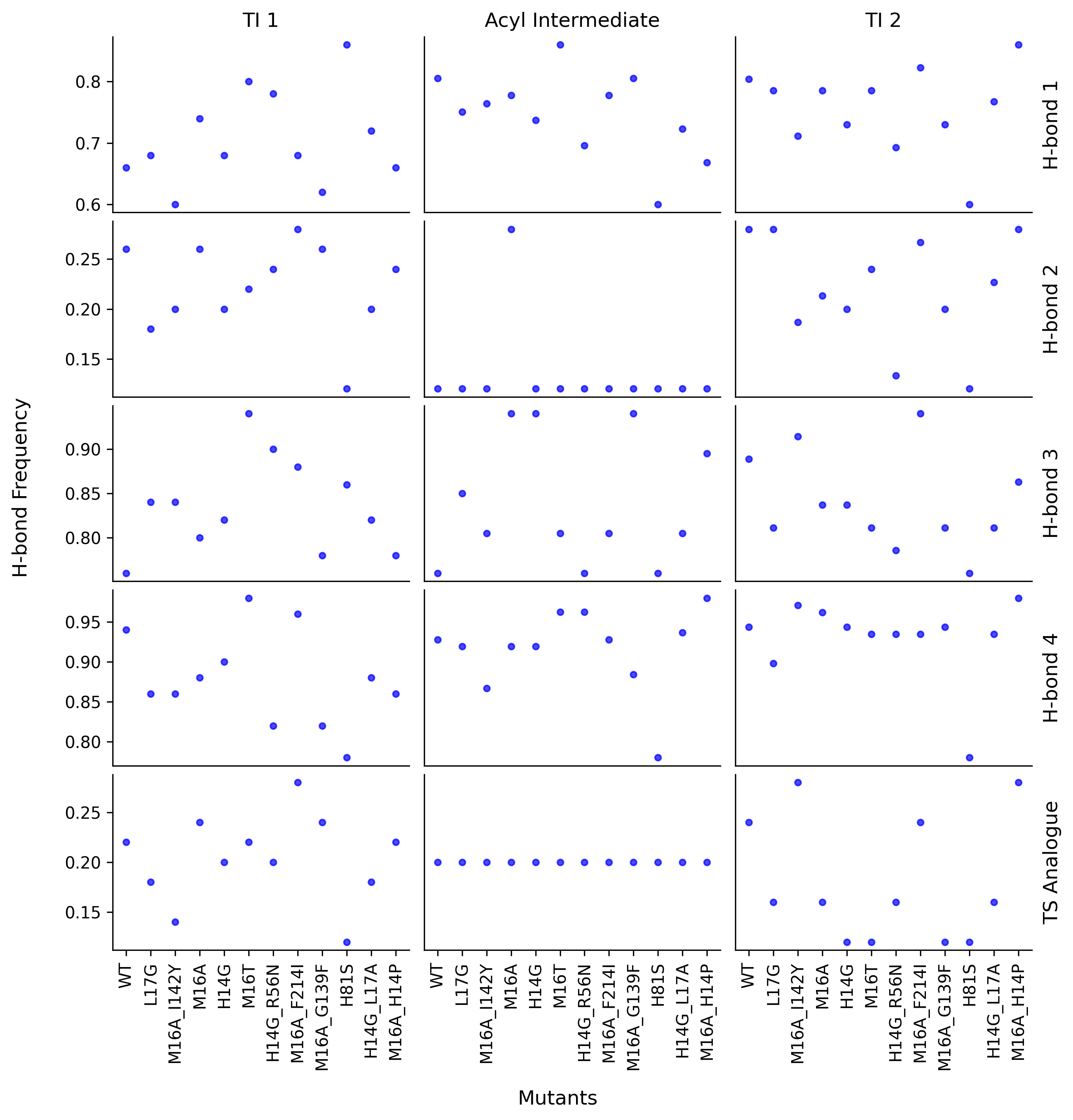


**Figure S4** *H-bond frequency in PLACER structures of Ser82 bound to p-NPB. Mutants are sorted by measured p-NPB activity from high to low (see Table 1).*

# References

1. Hehre WJ, Huang WW. Chemistry with computation: An introduction to SPARTAN: Wavefunction, Inc.; 1995.

2. Nardini M, Lang DA, Liebeton K, Jaeger K-E, Dijkstra BW. Crystal structure of pseudomonas aeruginosa lipase in the open conformation THE prototype for family I. 1 of bacterial lipases. Journal of Biological Chemistry. 2000;275(40):31219-25.

3. Shao Y, Molnar LF, Jung Y, Kussmann J, Ochsenfeld C, Brown ST, et al. Advances in methods and algorithms in a modern quantum chemistry program package. Physical Chemistry Chemical Physics. 2006;8(27):3172-91.

4. Wang J, Wang W, Kollman PA, Case DA. Antechamber: an accessory software package for molecular mechanical calculations. J Am Chem Soc. 2001;222:U403.

5. Jorgensen WL, Maxwell DS, Tirado-Rives J. Development and testing of the OPLS all-atom force field on conformational energetics and properties of organic liquids. Journal of the American Chemical Society. 1996;118(45):11225-36.

6. Krivov GG, Shapovalov MV, Dunbrack Jr RL. Improved prediction of protein side-chain conformations with SCWRL4. Proteins: Structure, Function, and Bioinformatics. 2009;77(4):778-95.

7. Hess B, Kutzner C, Van Der Spoel D, Lindahl E. GROMACS 4: algorithms for highly efficient, load-balanced, and scalable molecular simulation. Journal of Chemical Theory and Computation. 2008;4(3):435-47.

8. Lynch BJ, Truhlar DG. How well can hybrid density functional methods predict transition state geometries and barrier heights? The Journal of Physical Chemistry A. 2001;105(13):2936-41.

9. Rastelli G, Rio AD, Degliesposti G, Sgobba M. Fast and accurate predictions of binding free energies using MM‐PBSA and MM‐GBSA. Journal of Computational Chemistry. 2010;31(4):797-810.

10. Mobley DL, Bannan CC, Rizzi A, Bayly CI, Chodera JD, Lim VT, et al. Escaping atom types in force fields using direct chemical perception. Journal of Chemical Theory and Computation. 2018;14(11):6076-92.

11. Kent WJ. BLAT—the BLAST-like alignment tool. Genome Research. 2002;12(4):656-64.

12. Altschul SF, Madden TL, Schäffer AA, Zhang J, Zhang Z, Miller W, et al. Gapped BLAST and PSI-BLAST: a new generation of protein database search programs. Nucleic acids research. 1997;25(17):3389-402.

13. Jones DT. Protein secondary structure prediction based on position-specific scoring matrices. Journal of Molecular Biology. 1999;292(2):195-202.
