# Supplementary material for: Resolution of physics and deep learning-based protein engineering filters: A case study with a lipase for industrial substrate hydrolysis
Source: PLoS One. 2025 Sep 12;20(9):e0332409. doi: 10.1371/journal.pone.0332409 (PMC12431253; doi:10.1371/journal.pone.0332409)
Supplement: Attachment — Submitted filename: Point by Point Response.docx [file pone.0332409.s003.docx]

Point by Point Response

Journal Requirements:

When submitting your revision, we need you to address these additional requirements.

1. Please ensure that your manuscript meets PLOS ONE's style requirements, including those for file naming. The PLOS ONE style templates can be found at

<https://journals.plos.org/plosone/s/file?id=wjVg/PLOSOne_formatting_sample_main_body.pdf> and

🡪 Response: We updated the manuscript and filenames accordingly.

<https://journals.plos.org/plosone/s/file?id=ba62/PLOSOne_formatting_sample_title_authors_affiliations.pdf>

🡪 Response: We updated the title page accordingly.

2. Thank you for stating the following financial disclosure:
D.DC and S.G. were supported by the National Institute of General Medical Sciences of the National Institutes of Health under award number R15GM155803. D.DC. and P.D. thank the graduate school iGRASPseed for funding. Part of this study was supported by the Deutsche Forschungsgemeinschaft (DFG, German Research Foundation) through funding no. JA 448/8-1 to KEJ. G.F.S. and D.DC.


Please state what role the funders took in the study. If the funders had no role, please state: "The funders had no role in study design, data collection and analysis, decision to publish, or preparation of the manuscript."
If this statement is not correct you must amend it as needed.
Please include this amended Role of Funder statement in your cover letter; we will change the online submission form on your behalf.

🡪 Response: We updated the cover letter accordingly

3. Thank you for stating the following in the Acknowledgments Section of your manuscript:
D.DC and S.G. were supported by the National Institute of General Medical Sciences of the National Institutes of Health under award number R15GM155803. D.DC. and P.D. thank the graduate school

22 iGRASPseed for funding. Part of this study was supported by the Deutsche Forschungsgemeinschaft (DFG, German Research Foundation) through funding no. JA 448/8-1 to KEJ. G.F.S. and D.DC. gratefully acknowledge the computing time granted by the JARA-HPC Vergabegremium and VSR commission on the supercomputer JUROPA at Forschungszentrum Jülich.


We note that you have provided funding information that is not currently declared in your Funding Statement. However, funding information should not appear in the Acknowledgments section or other areas of your manuscript. We will only publish funding information present in the Funding Statement section of the online submission form.
Please remove any funding-related text from the manuscript and let us know how you would like to update your Funding Statement. Currently, your Funding Statement reads as follows:
D.DC and S.G. were supported by the National Institute of General Medical Sciences of the National Institutes of Health under award number R15GM155803. D.DC. and P.D. thank the graduate school iGRASPseed for funding. Part of this study was supported by the Deutsche Forschungsgemeinschaft (DFG, German Research Foundation) through funding no. JA 448/8-1 to KEJ. G.F.S. and D.DC.


Please include your amended statements within your cover letter; we will change the online submission form on your behalf.

🡪 Response: We updated the manuscript and cover letter accordingly.

4. Please remove all personal information, ensure that the data shared are in accordance with participant consent, and re-upload a fully anonymized data set.

🡪 Response: Not applicable.

Note: spreadsheet columns with personal information must be removed and not hidden as all hidden columns will appear in the published file.

🡪 Response: Not applicable.

Additional guidance on preparing raw data for publication can be found in our Data Policy (<https://journals.plos.org/plosone/s/data-availability#loc-human-research-participant-data-and-other-sensitive-data>) and in the following article: <http://www.bmj.com/content/340/bmj.c181.long>.

🡪 Response: Not applicable.

5. If the reviewer comments include a recommendation to cite specific previously published works, please review and evaluate these publications to determine whether they are relevant and should be cited. There is no requirement to cite these works unless the editor has indicated otherwise. 

🡪 Response: Not applicable.


6. Please review your reference list to ensure that it is complete and correct. If you have cited papers that have been retracted, please include the rationale for doing so in the manuscript text, or remove these references and replace them with relevant current references. Any changes to the reference list should be mentioned in the rebuttal letter that accompanies your revised manuscript. If you need to cite a retracted article, indicate the article’s retracted status in the References list and also include a citation and full reference for the retraction notice.

🡪 Response: We revied as requested.

Additional Editor Comments:

Thank you very much for submitting your manuscript to PLOS One. Your manuscript has now been reviewed by experts in the field. Please revise the manuscript according to the referees' comments and upload the revised file.


Reviewers' comments:

Reviewer's Responses to Questions

Comments to the Author

1. Is the manuscript technically sound, and do the data support the conclusions?

The manuscript must describe a technically sound piece of scientific research with data that supports the conclusions. Experiments must have been conducted rigorously, with appropriate controls, replication, and sample sizes. The conclusions must be drawn appropriately based on the data presented. 

Reviewer #1: Yes

2. Has the statistical analysis been performed appropriately and rigorously? 

Reviewer #1: Yes

3. Have the authors made all data underlying the findings in their manuscript fully available?

The PLOS Data policy requires authors to make all data underlying the findings described in their manuscript fully available without restriction, with rare exception (please refer to the Data Availability Statement in the manuscript PDF file). The data should be provided as part of the manuscript or its supporting information, or deposited to a public repository. For example, in addition to summary statistics, the data points behind means, medians and variance measures should be available. If there are restrictions on publicly sharing data—e.g. participant privacy or use of data from a third party—those must be specified.

Reviewer #1: Yes

4. Is the manuscript presented in an intelligible fashion and written in standard English?

PLOS ONE does not copyedit accepted manuscripts, so the language in submitted articles must be clear, correct, and unambiguous. Any typographical or grammatical errors should be corrected at revision, so please note any specific errors here.

Reviewer #1: No

5. Review Comments to the Author

Please use the space provided to explain your answers to the questions above. You may also include additional comments for the author, including concerns about dual publication, research ethics, or publication ethics. (Please upload your review as an attachment if it exceeds 20,000 characters)

Reviewer #1: This study focuses on evaluating the limitations of enzyme design methods through computation in optimizing enzyme activity against non-natural substrates, specifically using the LipA enzyme from Pseudomonas aeruginosa to hydrolyse the Roche ester substrate. There are some of my inputs that can improve the quality of this article.

🡪 Response: We thank the reviewer for his insightful comments and positive assessment of our work. Detail response follows below.

1) The title is very general. please be more specific

🡪 Response: We extended the title with the following details: **Resolution of physics and deep learning-based protein engineering filters: A case study with a lipase for industrial substrate hydrolysis**

2) Please specify the selection of H14/M16/L17/H81!

🡪 Response: We have added clarifying text to the main manuscript describing the selection criteria for these specific residues: "We used an ensemble-based simulation protocol (Supplementary Information S6) to select promising single point candidate mutations in presence of Roche ester at residues H14, M16, L17, and H81, all located near the active site on flexible loop regions (Fig. 2A). These four variants were selected from 669 computationally viable mutations based on predicted transition state stabilization (ΔẼ < 0.985), evolutionary conservation (PSSM > -4.0), and proximity to substrate (<5 Å)." The detailed computational protocol is provided in Supplementary Information S6, as our focus here is on evaluating the performance of different computational approaches in distinguishing these selected variants rather than extensively justifying the initial selection process.

3) Compare the RMSF (or RMSD relative to the crystal structure) in the CONCOORD ensemble vs. MD 100 ns. In this article, CONCOORD is mentioned to “lower the pressure” due to constraints. However, doesn't this make the ensemble too “too constrained”, thus losing millisecond-scale motions that can affect binding?

🡪 Response: We agree this is an important consideration. As shown in Figure 2B, the CONCOORD ensemble captures RMSF values comparable to those observed in 100 ns MD simulations, indicating that local flexibility around the active site is reasonably preserved. While CONCOORD cannot capture millisecond-scale motions, our approach specifically constrains the transition state geometry to focus on the immediate catalytic environment during the chemical reaction step. For enzyme catalysis, the critical timescales are typically microseconds to milliseconds for the chemical step itself, rather than the longer conformational transitions that might affect substrate binding. Importantly, the experimental validation demonstrates that variants selected using this constrained ensemble approach do exhibit significant differences in catalytic activity, suggesting that the method captures the relevant structural features despite its limitations. A more comprehensive comparison of ensemble methods and their ability to predict catalytic differences would be valuable future work, but falls outside the scope of this study focused on evaluating current computational activity filters.

4) The author does not provide an explanation of the computational methods used, for example, AMBER99SB-ILDN and M06-2X/6-31G. Please clarify!

🡪 Response: We checked the computational methods for lacking descriptions throughout and added the following explanations:

AMBER99SB-ILDN is a widely validated protein force field incorporating refined side-chain torsion parameters for isoleucine, leucine, aspartate, and asparagine residues. These adjustments improve the accuracy of side-chain dynamics, which can enhance modeling of hydrophobic contacts and packing, such as those critical in lipase active sites.

M06-2X/6-31G(d,p) DFT calculations were performed using Gaussian 16 (67). The M06-2X functional is a hybrid meta-GGA method incorporating 54% Hartree–Fock exchange, developed for accurate main-group thermochemistry, kinetics, and noncovalent interactions. The 6-31G(d,p) split-valence double-zeta basis set includes polarization functions (d on heavy atoms, p on hydrogens), which improve the description of electron distribution changes during bond formation and cleavage in the enzymatic reaction.

5) Please specify the solvent conditions in the DFT calculations!

🡪 Response: Thank you for bringing this to our attention. We added the following clarification to the methods: No implicit or explicit solvent model was applied; all DFT calculations were performed in the gas phase.

6) In Figure 5, what specific criteria (distance & angle) were used to detect H-bonds in the analysis? Were other thresholds (eg: 3.2 Å) tested to see if the pattern changed?

🡪 Response: The hydrogen bond analysis in Figure 5 employed standard geometric criteria implemented in the MDAnalysis package: a donor-acceptor distance cutoff of 3.0 Å and a donor-hydrogen-acceptor angle cutoff of 150°. To address potential sensitivity to these parameters, we tested alternative distance thresholds ranging from 2.5 to 4.0 Å. While these different cutoffs shifted the overall distribution of hydrogen bond frequencies, they did not reveal any meaningful patterns correlating with enzymatic activity - the distributions remained essentially random across all tested parameters. This reinforces our conclusion that hydrogen bond persistence, at least as captured by standard geometric criteria, does not serve as a reliable predictor of catalytic efficiency differences between these lipase variants. We have added this detail to the revised manuscript.


While revising your submission, please upload your figure files to the Preflight Analysis and Conversion Engine (PACE) digital diagnostic tool, <https://pacev2.apexcovantage.com/>. PACE helps ensure that figures meet PLOS requirements. To use PACE, you must first register as a user. Registration is free. Then, login and navigate to the UPLOAD tab, where you will find detailed instructions on how to use the tool. If you encounter any issues or have any questions when using PACE, please email PLOS at [figures@plos.org](mailto:figures@plos.org). Please note that Supporting Information files do not need this step.

🡪 Response: We used the PACE tool to create .tiff files of all figures. This worked remarkably well.
